# Attenuation of cerebral edema facilitates recovery of glymphatic system function after status epilepticus

**DOI:** 10.1172/jci.insight.151835

**Published:** 2021-09-08

**Authors:** Kewei Liu, Juan Zhu, Yuan Chang, Zhenzhou Lin, Zhu Shi, Xing Li, Xing Chen, Chuman Lin, Suyue Pan, Kaibin Huang

**Affiliations:** 1 Department of Neurology, Nanfang Hospital, Southern Medical University, Guangzhou, China.; 2 Department of Neurology, Dongguan Hospital, Southern Medical University, Dongguan, China.; 3 Department of Neurology, Guangzhou First People’s Hospital, School of Medicine, South China University of Technology, Guangzhou, Guangdong, China.; 4 Department of Neurology, Zhujiang Hospital, Southern Medical University, Guangzhou, Guangdong, China.

**Keywords:** Neuroscience, Therapeutics, Epilepsy, Neurodegeneration

## Abstract

Status epilepticus (SE) is a neurological emergency usually accompanied by acute cerebral edema and long-term cognitive impairment, and is characterized by neurodegeneration and aberrant hyperphosphorylated tau protein (p-tau) aggregation. The glia-lymphatic (glymphatic) system plays a central role in facilitating the clearance of metabolic waste from the brain, but its relationship with cerebral edema and cognitive dysfunction after SE is unclear. We hypothesized that cerebral edema after SE might impair glymphatic system function through compression, thus leading to impaired removal of metabolic waste, and ultimately affecting long-term cognitive function. Our results showed that glymphatic system function was temporarily impaired, as evidenced by 2-photon imaging, MRI enhancement, imaging of brain sections, and astrocytic water channel aquaporin 4 (AQP4) protein polarization. The severity of cerebral edema on MRI correlated well with glymphatic system dysfunction within 8 days following SE. Moreover, when cerebral edema was alleviated by glibenclamide treatment or genetic deletion of *Trpm4*, post-SE glymphatic system function recovered earlier, along with fewer p-tau–deposited neurons and neuronal degeneration and better cognitive function. These findings suggest that SE-induced cerebral edema may cause glymphatic system dysfunction and render the post-SE brain vulnerable to p-tau aggregation and neurocognitive impairment.

## Graphical abstract

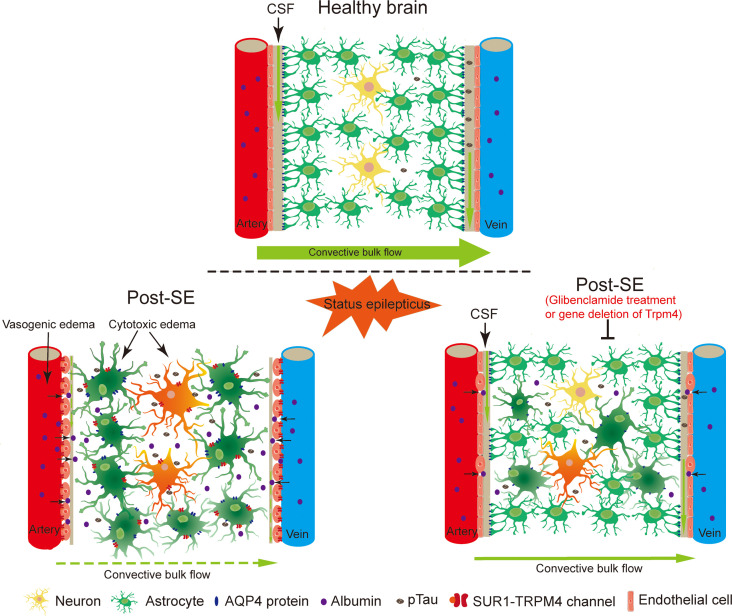

## Introduction

Status epilepticus (SE) is a neurological emergency characterized by sustained epileptic activity or frequent seizures without return to baseline, with high mortality and morbidity (1). Even after the cessation of SE, a significant proportion of patients have a persistent neurocognitive impairment (2, 3). In animal studies of SE, prolonged seizures usually result in significant cognitive deficits (4, 5). In addition to the direct neuronal damage caused by SE, deposition of hyperphosphorylated tau protein (p-tau) is thought to cause neurodegeneration after SE (6). Furthermore, the degree of p-tau pathology correlates with the severity of cognitive decline (7). However, the mechanism of aberrant p-tau aggregation after SE remains unclear.

The recently discovered glia-lymphatic (glymphatic) system, which consists of a brain-wide perivascular space (PVS), supports the exchange of interstitial fluid (ISF) and cerebrospinal fluid (CSF), and facilitates the clearance of interstitial solutes and metabolic waste products (e.g., p-tau and β-amyloid) from the brain parenchyma (8). Glymphatic system dysfunction is present in various neurodegenerative or epilepsy-associated diseases, including Alzheimer’s (9), aging (10), traumatic brain injury (11), and stroke (12). However, it remains unclear whether SE causes glymphatic system dysfunction and thereby results in aberrant p-tau aggregation.

Cerebral edema is almost inevitable after SE and correlates with poor prognosis (13). Theoretically, both cytotoxic edema and vasogenic edema may squeeze the PVS through a space-occupying effect to disturb the glymphatic system function, thus affecting the normal clearance of p-tau. Until now, only 1 study in 1990 reported that focal vasogenic edema could block tracer movement through PVS in cat brain (14). Here, we hypothesized that SE-induced cerebral edema during the acute stage may interfere with glymphatic system function, leading to the deposition of p-tau and long-term cognitive impairment after SE.

To test this hypothesis, we investigated the dynamic changes in glymphatic system function in a mouse model of SE, and the relationship between the impaired glymphatic system and cerebral edema. We found that glymphatic system function was impaired 1 day after SE, continued to worsen until day 3, and partially recovered on day 8. Meanwhile, the development of cerebral edema correlated well with glymphatic system dysfunction within 8 days following SE. Moreover, when cerebral edema was alleviated by glibenclamide treatment or genetic deletion of *Trpm4*, post-SE glymphatic system function recovered earlier, along with fewer p-tau–deposited neurons and neuronal degeneration and better cognitive function. These findings suggest that glymphatic system function was temporarily impaired after SE, and might be caused by post-SE cerebral edema. The intervention of cerebral edema may favor the recovery of glymphatic system function and improve cognitive impairment related to the deposition of p-tau after SE.

## Results

### Glymphatic system function was temporarily impaired after SE.

**Figure 1:**
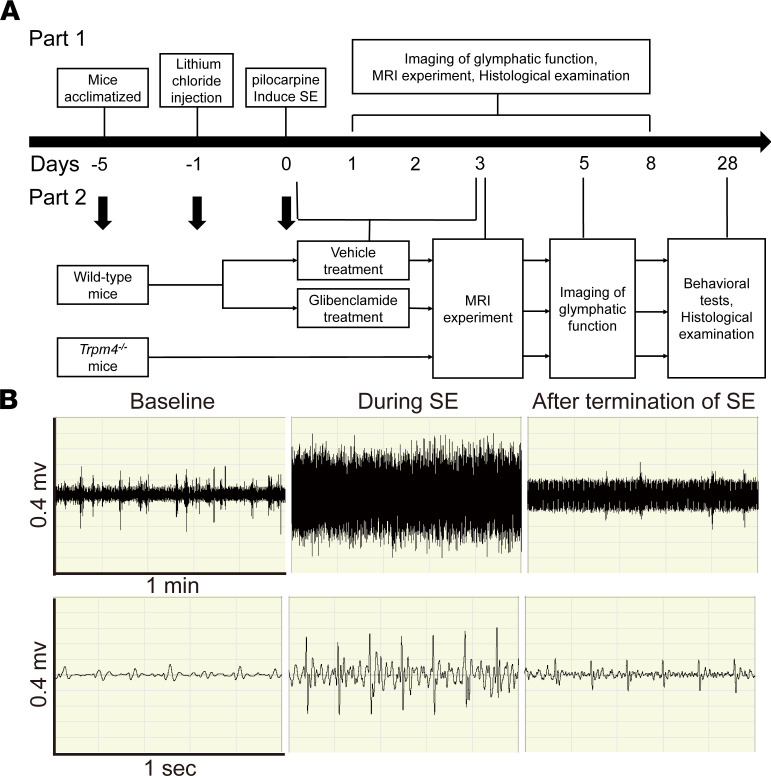
Flow diagram and EEG. (**A**) Flow diagram for the study assessments on a timeline. (**B**) Representative EEG traces.

We used the lithium-pilocarpine–induced SE model in this study, and the success of SE modeling was confirmed by EEG (Figure 1, A and B). The characteristics of EEG during SE were high-frequency and high-amplitude discharges. Of the 278 mice that successfully induced SE, 38 died before predefined time points, 200 successfully terminated their seizures with a single dose of diazepam, and 40 required a second dose of diazepam. After the termination of behavioral seizures by diazepam, the high-frequency and high-amplitude discharges were profoundly reduced.

**Figure 2:**
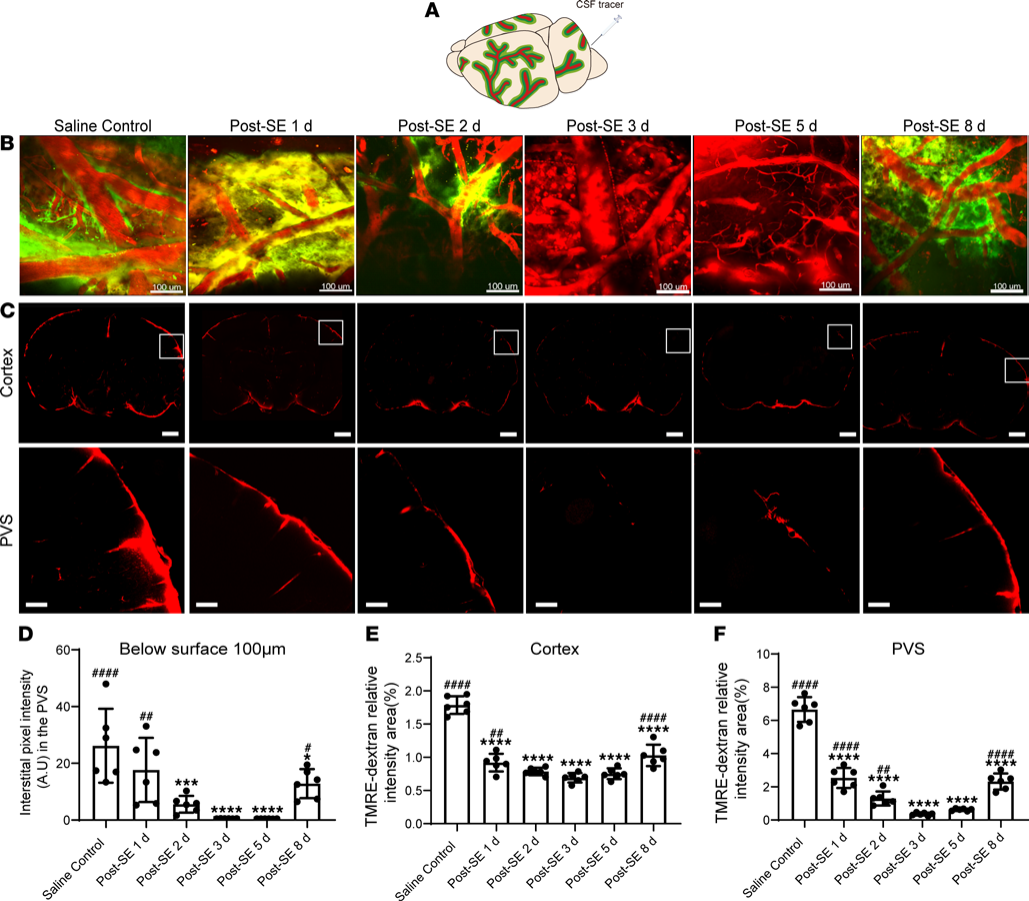
Glymphatic system function is temporarily impaired after SE. (**A**) Schematic depiction of intracisternal administration of CSF tracer. (**B**) Representative pseudo-3D images of the cerebral vasculature and the distribution of the tracer (observation location: 100 μm below brain surface). Red, cerebral vasculature; green, cerebrospinal fluid tracer. Scale bars: 100 μm. (**C**) Representative images (100 μm thick, observation location: bregma −1 mm) of fluorescence tracer (red, TMRE-dextran) in coronal brain sections (upper panel) and higher-magnification images for the PVS (lower panel, derived from boxes in upper panel). Scale bars: 1000 μm (upper panel) and 200 μm (lower panel). (**D**) Quantitative analysis of the mean pixel intensity of the tracer in the pseudo-3D images (*n* = 6 each). (**E** and **F**) Quantitative analysis of the percentage area of fluorescence in the whole brain section and PVS (*n* = 6 each). Data are shown as the mean ± SD. Differences within multiple groups were examined by 1-way ANOVA with Bonferroni’s multiple comparison test. *P < 0.05, ***P < 0.001, ****P < 0.0001 versus saline control group; ^#^*P* < 0.05, ^##^*P* < 0.01, ^####^*P* < 0.0001 versus 3 days after SE.

We first evaluated the glymphatic system function after SE via in vivo 2-photon imaging at 1, 2, 3, 5, and 8 days. In the saline control group, the CSF tracer moved into the cortex along cerebrum-penetrating arterioles, spreading through the surrounding interstitium on the surface of the cortex (Figure 2B). Two days after SE, the tracer influx was significantly decreased and only appeared around a small number of cerebrum-penetrating arterioles (Figure 2, B and D; *P* = 0.0002 vs. saline control). Notably, the CSF tracer influx virtually disappeared 3 days after SE and remained undetectable 5 days after SE (Figure 2, B and D; *P* < 0.0001 vs. saline control). Eight days after SE, the tracer influx was partially recovered (Figure 2, B and D; *P* = 0.0364 vs. 3 days after SE). These results indicate that the glymphatic system function continued to decline after SE reached the lowest point at 3 days after SE, and partially recovered at 8 days after SE.

Although 2-photon imaging allows in vivo observation of the glymphatic system function, only the superficial region of the brain through a small window (approximately 3 mm in diameter) is available for observation (15). Therefore, we further evaluated the brain-wide glymphatic system function ex vivo by slowly injecting 6 μL of CSF tracer (70 kDa tetramethylrhodamine-dextran [TMRE-dextran]) into the cisterna magna and observed the penetration of CSF tracer. In the saline control group, whole-slice fluorescence imaging demonstrated that CSF tracer surrounded the edges of brain sections, and higher-magnification images of the PVS showed that the tracer existed in the PVS (Figure 2C). At 1 day after SE, the intensity of the tracer was significantly decreased at the edge of brain sections, and only a small amount of tracer appeared in the PVS. At 3 days after SE, only the bottom of the brain sections exhibited a small amount of tracer, and the tracer substantially vanished in the PVS-surrounding vessels (Figure 2, E and F; *P* < 0.0001 vs. saline control). The tracer influx was partially recovered 8 days after SE (Figure 2, E and F; *P* < 0.0001 vs. 3 days after SE), consistent with the 2-photon imaging results. These results indicate that glymphatic system function was already impaired 1 day after SE, became worse 3 days after SE, and partially recovered 8 days after SE. Compared with the findings from 2-photon imaging, less fluorescence existed in the PVS via ex vivo imaging, probably due to perfusion fixation with 4% paraformaldehyde (PFA) causing abnormal retrograde flow and shrinking the PVS (16).

**Figure 3:**
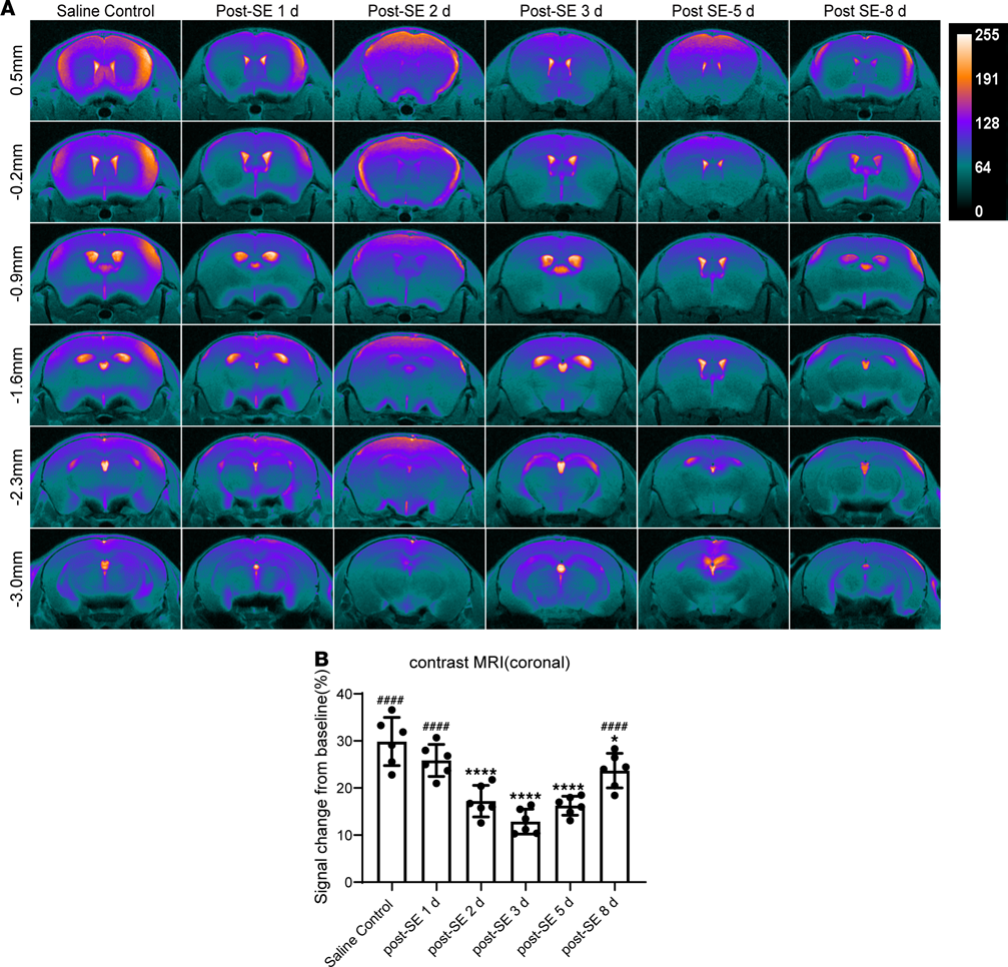
Glymphatic system function is temporarily impaired after SE, as demonstrated by contrast-enhanced MRI (coronal). (**A**) Representative pseudocolor images showing Gd-DTPA distribution in coronal brain sections from bregma +0.5 mm to −3.0 mm. (**B**) Quantitative analysis of signal change percentage in the coronal plane (*n* = 6 each). Data are shown as the mean ± SD. Differences within multiple groups were examined by 1-way ANOVA with Bonferroni’s multiple comparison test. *P < 0.05, ****P < 0.0001 versus saline control group; ^####^*P* < 0.0001 versus 3 days after SE.

**Figure 4:**
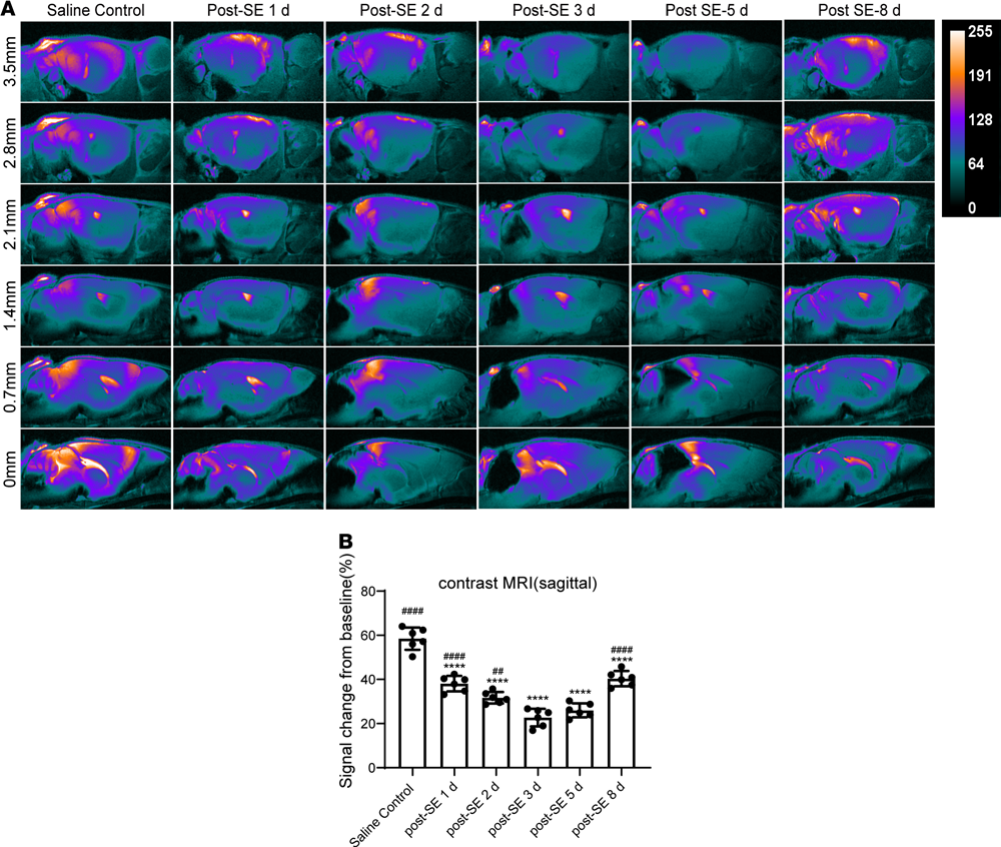
Glymphatic system function is temporarily impaired after SE, as demonstrated by contrast-enhanced MRI (sagittal). (**A**) Representative pseudocolor images showing Gd-DTPA in sagittal brain sections from lateral +3.5 mm to −3.5 mm (half of the representative pictures are shown). (**B**) Quantitative analysis of signal change percentage in the sagittal plane (*n* = 6 each). Data are shown as the mean ± SD. Differences within multiple groups were examined by 1-way ANOVA with Bonferroni’s multiple comparison test. ****P < 0.0001 versus saline control group; ^##^*P* < 0.01, ^####^*P* < 0.0001 versus 3 days after SE.

Ex vivo imaging of glymphatic system function via perfusion-fixation might alter the normal flow direction and cause a 10-fold reduction in PVS size, thereby biasing the actual results (16). Hence, we performed contrast-enhanced MRI to confirm the above results in anesthetized mice. We evaluated the signal change in 2 planes after injection of the contrast enhancer gadolinium–diethylenetriamine pentaacetic acid (Gd-DTPA) into the cisterna magna and added a pseudocolor to the images. In the coronal plane, we selected 6 sections from bregma +0.5 mm to −3.0 mm, and calculated the Gd-DTPA signal intensity ratio (defined by the percentage signal change from the baseline). For the mice in the saline control group, Gd-DTPA appeared in the lateral ventricles and diffused into the brain parenchyma (Figure 3A). For the mice in the SE group, the signal intensity ratio was significantly decreased starting 2 days after SE (Figure 3B; *P* < 0.0001 vs. saline control), reached a nadir 3 days after SE (Figure 3B; *P* < 0.0001 vs. saline control), and gradually recovered 8 days after SE (Figure 3B; *P* < 0.0001 vs. 3 days after SE). In the sagittal plane, we selected 11 sections from bregma +3.5 mm to −3.5 mm (half of the representative pictures are shown) and observed similar results to those of the coronal plane (Figure 4, A and B). In general, these data, consistent with the results above, indicate that the decline in glymphatic system function began 1 day after SE, reached the lowest point 3 days after SE, and partially recovered 8 days after SE.

### The efflux of ISF from the brain parenchyma was temporarily impaired after SE.

**Figure 5:**
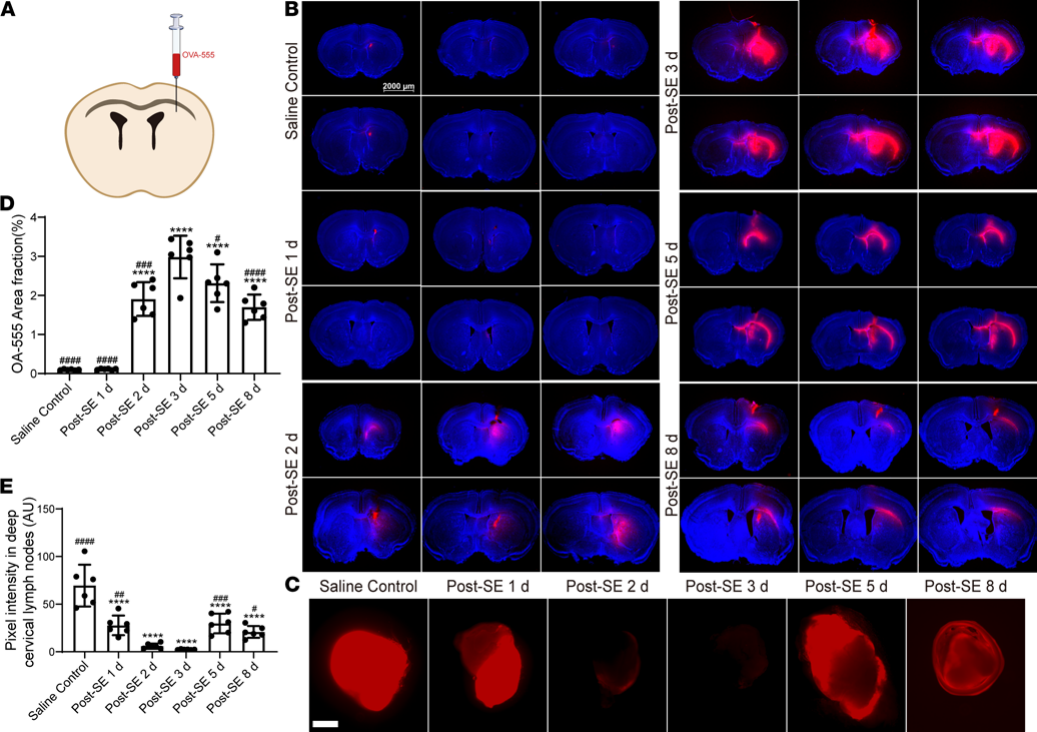
Interstitial solute drainage is temporarily impaired after SE. (**A**) Schematic depiction of intrastriatal injection of OVA-555 (+1.5 mm AP, +1.5 mm ML, –2.5 mm DV from bregma). (**B**) Representative images of OVA-555 residue in the brain (6 brain sections). Scale bar: 2000 μm. (**C**) Representative images of the fluorescence intensity of OVA-555 drainage into the dCLNs. Scale bar: 500 μm. (**D**) Quantitative analysis of the percentage area of fluorescence in the 6 brain sections (*n* = 6 each). (**E**) Quantitative analysis of fluorescence intensity in the dCLNs (*n* = 6 each). Data are shown as the mean ± SD. Differences within multiple groups were examined by 1-way ANOVA with Bonferroni’s multiple comparison test. ****P < 0.0001 versus saline control group; ^#^*P* < 0.05, ^##^*P* < 0.01, ^###^*P* < 0.001, ^####^*P* < 0.0001 versus 3 days after SE.

We next examined whether the efflux of ISF from the brain parenchyma was altered after SE. Two hours after the intraparenchymal injection of OA-555, the efflux of OA-555 was assessed in the parenchyma and deep cervical lymph nodes (dCLNs) of mice. Almost no red fluorescence was observed in the parenchyma in the saline control group, indicating that most interstitial molecules had been removed (Figure 5B). After SE, however, the red fluorescence started to increase on day 2 and became more evident on day 3 (Figure 5D; *P* < 0.0001 vs. saline control). The red fluorescence weakened 5 days after SE (Figure 5D; *P* = 0.0187 vs. 3 days after SE) and further decreased 8 days after SE (Figure 5D; *P* < 0.0001 vs. 3 days after SE).

Lymphatic efflux of parenchymal or ISF solutes primarily drains into dCLNs (17). In the saline control group, the red fluorescence in the dCLNs was intensely bright, indicating that most interstitial molecules injected into the parenchyma were discharged into the dCLNs (Figure 5C). However, the fluorescence intensity in the dCLNs was significantly decreased starting 1 day after SE and substantially vanished 3 days after SE (Figure 5E; both *P* < 0.0001 vs. saline control). The fluorescence intensity was partially recovered at 5 and 8 days after SE (Figure 5E; *P* = 0.0009 and 0.0375 vs. 3 days after SE).

These results demonstrate that the ISF efflux began to decline 1 day after SE, was almost completely lost 3 days after SE, and began to improve 5 days after SE, indicating that the efflux of ISF was temporarily impaired after SE and recovered earlier than the CSF tracer influx.

### The expression and depolarization of AQP4 was temporarily increased after SE.

**Figure 6:**
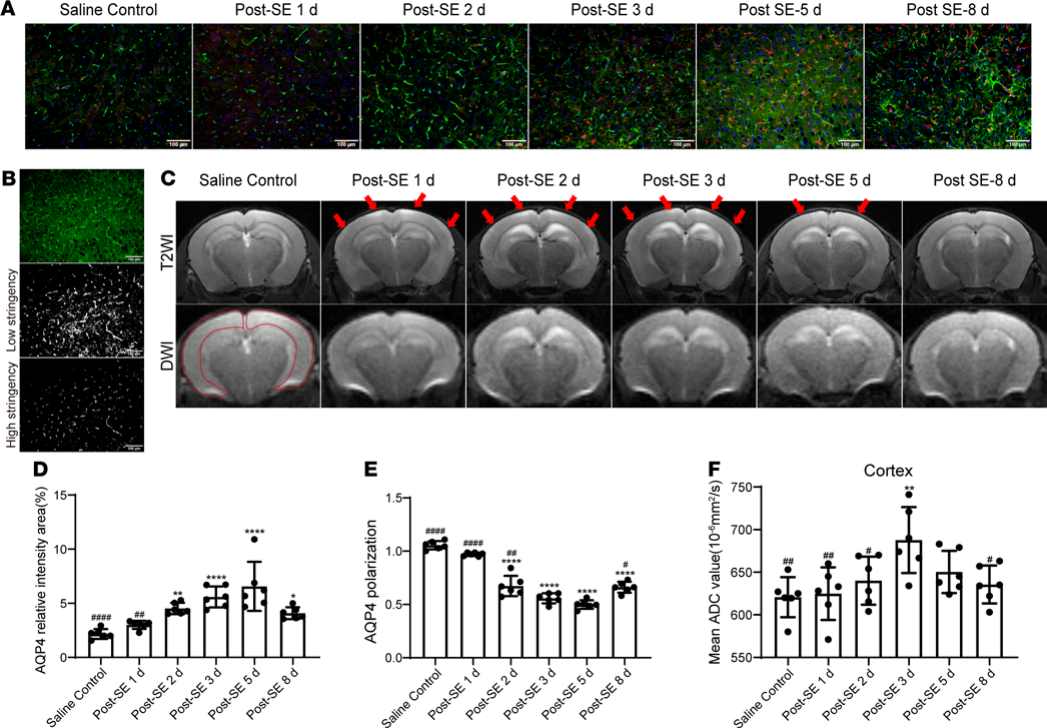
Analysis of aquaporin 4 (AQP4) expression and polarization, and cerebral edema after SE. (**A**) Representative images of AQP4 expression in the cortex (red, GFAP; green, AQP4; blue, DAPI). Scale bars: 100 μm. (**B**) Changes in AQP4 polarization were measured by image analysis. Shown are representative images of AQP4 polarization in the cortex 5 days after SE used for the calculation of AQP4 polarization. (**C**) Representative T2WI and DWI images. The red arrows indicate hyperintense T2WI in the bilateral parietal cortex; the red irregular area indicates calculated regions of interest of mean ADC values. (**D**) Quantitative analysis of AQP4 expression percentage in cortex (*n* = 6 each). (**E**) Quantitative analysis of AQP4 polarization in cortex (*n* = 6 each). (**F**) Quantitative analysis of mean ADC values of the bilateral cortex (*n* = 6 each). Data are shown as the mean ± SD. Differences within multiple groups were examined by 1-way ANOVA with Bonferroni’s multiple comparison test. *P < 0.05, **P < 0.01, ****P < 0.0001 versus saline control group; ^#^*P* < 0.05, ^##^*P* < 0.01, ^####^*P* < 0.0001 versus 3 days after SE.

Glymphatic system function has been reported to be highly dependent on the polarization of glial AQP4 channels (8). Thus, we evaluated the expression and polarization of AQP4 at different time points after SE (Figure 6A). Compared with saline control, the expression of AQP4 was significantly increased starting 2 days after SE (*P* = 0.0036 vs. saline control), reached the peak 5 days after SE (*P* < 0.0001 vs. saline control), and began to decrease 8 days after SE (*P* = 0.0021 vs. 5 days after SE) (Figure 6D). Meanwhile, the polarization of AQP4 was significantly reduced starting 2 days after SE (*P* < 0.0001 vs. saline control), reached the lowest level 5 days after SE (*P* < 0.0001 vs. saline control), and partially recovered 8 days after SE (*P* = 0.0112 vs. 5 days after SE) (Figure 6E). These results further support the temporary impairment of glymphatic system function after SE from the perspective of the polarization of AQP4 channels, which was impaired starting 2 days after SE, continued to worsen until 5 days after SE, and partially recovered on day 8 after SE.

### The severity of cerebral edema is consistent with the functional state of the glymphatic system after SE.

Researchers have speculated that the glymphatic system is related to vasogenic edema (8). We therefore performed cranial MRI to visualize and quantify cerebral edema after SE. T2-weighted imaging (T2WI) and diffusion-weighted imaging (DWI) changes were observed at different time points after SE. In the T2WI, post-SE mice showed a high signal in the bilateral parietal cortex (red arrows) starting on day 1, and the high signal was most apparent on day 3 and persisted until day 5 (Figure 6C). In apparent diffusion coefficient (ADC) images reconstructed from DWI, we evaluated the mean ADC value of the bilateral cortex. The mean ADC value was significantly increased 3 days after SE (Figure 6F; *P* = 0.0016 vs. saline control) and declined 8 days after SE (Figure 6F; *P* = 0.0182 vs. 3 days after SE). The high signal in T2WI and increased ADC values together indicate the formation of vasogenic edema in the bilateral cortex after SE (18), which reached the peak 3 days after SE and was partially alleviated 8 days after SE. The severity of cerebral edema was consistent with the functional state of the glymphatic system, which was damaged most severely 3 days after SE and recovered partially 8 days after SE, suggesting a potential correlation between glymphatic system dysfunction and cerebral edema.

### Glymphatic system function recovered earlier after intervention for cerebral edema.

**Figure 7:**
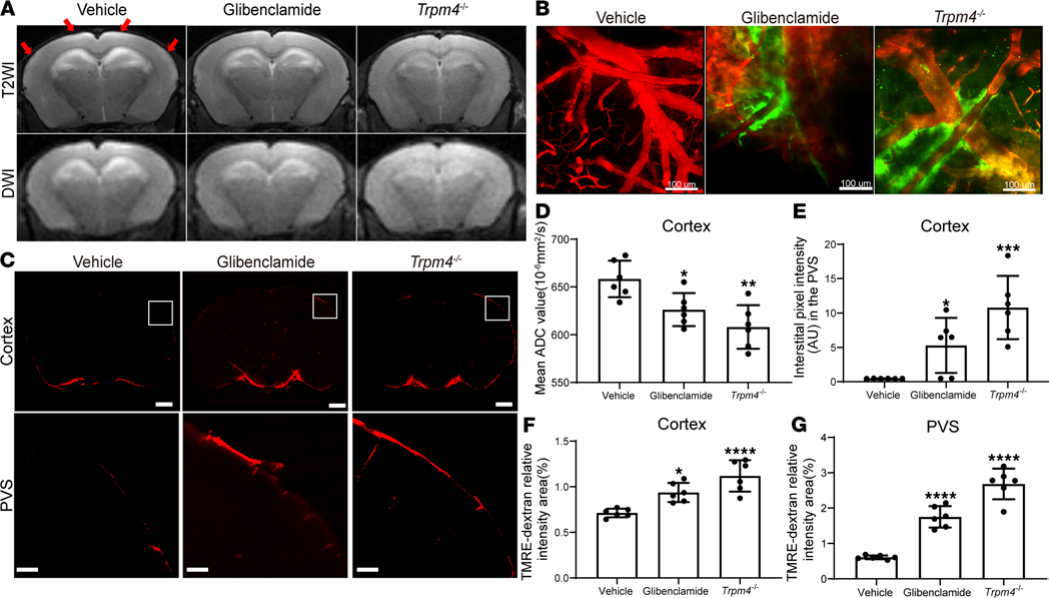
Analysis of cerebral edema 3 days after SE and glymphatic system function 5 days after SE, after genetic deletion of *Trpm4* and glibenclamide intervention. (**A**) Representative T2WI and DWI images. The red arrows indicate hyperintense T2WI in the bilateral parietal cortex. (**B**) Representative pseudo-3D images of the cerebral vasculature and the distribution of the tracer (red, cerebral vasculature; green, cerebrospinal fluid tracer). Scale bars: 100 μm. (**C**) Representative images (100 μm thick; observation location: bregma −1 mm) of fluorescence tracer (red, TMRE-dextran) in coronal brain sections (upper panel) and higher-magnification images for PVS (lower panel, derived from boxes in upper panel). Scale bars: 1000 μm (upper panel) and 200 μm (lower panel). (**D**) Quantitative analysis of mean ADC values of the bilateral cortex (*n* = 6 each). (**E**) Quantitative analysis of the mean pixel intensity of the tracer in the pseudo-3D images (*n* = 6 each). (**F** and **G**) Quantitative analysis of the percentage area of fluorescence in the whole brain section and PVS (*n* = 6 each). Data are shown as the mean ± SD. Differences within multiple groups were examined by 1-way ANOVA with Bonferroni’s multiple comparison test. *P < 0.05, **P < 0.01, ***P < 0.001, ****P < 0.0001 versus vehicle group.

To further explore the correlation between impaired glymphatic system function and cerebral edema, we used *Trpm4*-knockout (*Trpm4^–/–^*) mice and glibenclamide treatment to alleviate cerebral edema after SE. Consistent with our previous studies (5, 19), the mean ADC value was significantly lower in the glibenclamide (*P* = 0.0277 vs. vehicle group) and *Trpm4^–/–^* groups (*P* = 0.0011 vs. vehicle group) than in the vehicle group 3 days after SE (Figure 7, A and D). These results suggest that cerebral edema induced by SE was substantially attenuated by glibenclamide treatment and genetic deletion of *Trpm4*.

We evaluated the influx function of the glymphatic system via 2-photon imaging and imaging of brain sections after intervention with glibenclamide and *Trpm4* knockout. Although cerebral edema was markedly alleviated 3 days after SE after glibenclamide and *Trpm4^–/–^* interventions, glymphatic function had yet to recover at this time point. We found that CSF tracer influx was significantly increased in both the glibenclamide (*P* = 0.0146 vs. vehicle group) and *Trpm4^–/–^* groups (*P* = 0.0003 vs. vehicle group) 5 days after SE via 2-photon imaging (Figure 7, B and E). Consistently, tracers at the edge of brain sections and the PVS-surrounding vessels were significantly recovered in the glibenclamide and *Trpm4^–/–^* groups (Figure 7, C, F, and G).

**Figure 8:**
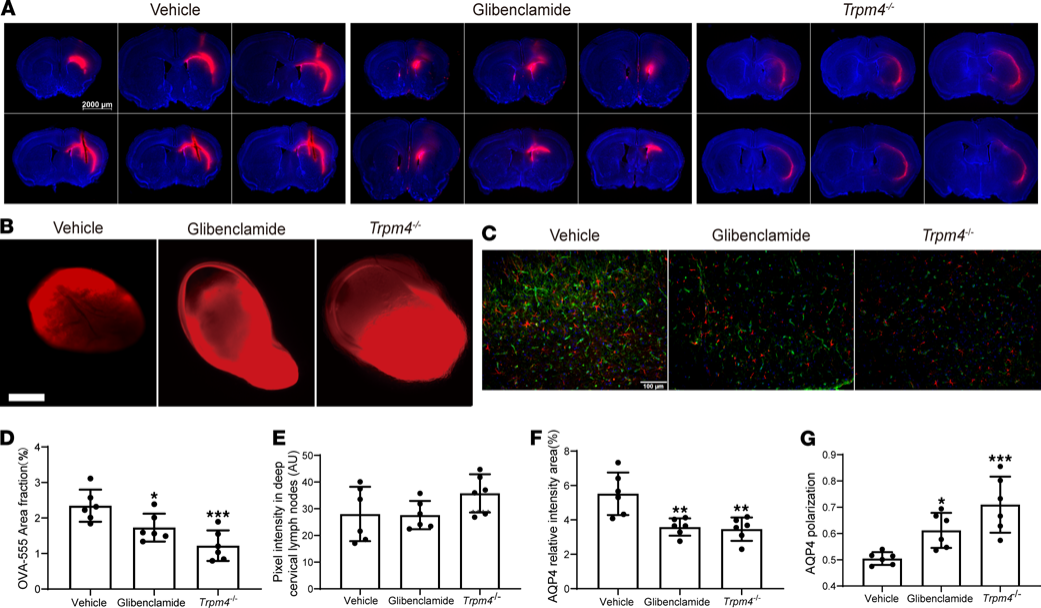
Analysis of interstitial solute drainage 5 days after SE, after genetic deletion of *Trpm4* and glibenclamide intervention. (**A**) Representative images of the OVA-555 residue in the brain (6 brain sections). Scale bar: 2000 μm. (**B**) Representative images of the fluorescence intensity of the OVA-555 drainage into the dCLNs. Scale bar: 500 μm. (**C**) Representative images of AQP4 expression in the cortex (red, GFAP; green, AQP4; blue, DAPI). Scale bar: 100 μm. (**D**) Quantitative analysis of the percentage area of fluorescence in the 6 brain sections (*n* = 6 each). (**E**) Quantitative analysis of fluorescence intensity in the dCLNs (*n* = 6 each). (**F**) Quantitative analysis of AQP4 expression percentage in the cortex (*n* = 6 each). (**G**) Quantitative analysis of AQP4 polarization in the cortex (*n* = 6 each). Data are shown as the mean ± SD. Differences within multiple groups were examined by 1-way ANOVA with Bonferroni’s multiple comparison test. *P < 0.05, **P < 0.01, ***P < 0.001 versus vehicle group.

Next, we examined the efflux of ISF after cerebral edema intervention (Figure 8A). Compared with the vehicle-treated group, the residual dye in brain sections was significantly decreased in the glibenclamide (Figure 8D; *P* = 0.0476 vs. vehicle group) and *Trpm4^–/–^* groups (Figure 8D; *P* = 0.0007 vs. vehicle group). However, the fluorescence intensity in the dCLNs showed no significant difference in the glibenclamide and *Trpm4^–/–^* groups compared with the vehicle group (Figure 8, B and E), which may be because the fluorescence intensity of dCLNs in each group was too strong.

We also assessed AQP4 expression and depolarization after the interventions (Figure 8C). Compared with the vehicle-treated group, AQP4 expression and depolarization were significantly decreased in the glibenclamide and *Trpm4^–/–^* groups (Figure 8, F and G). These results further support the improvement of glymphatic system function after the intervention for cerebral edema.

Taken together, these data suggest that cerebral edema alleviation significantly promotes the earlier recovery of impaired glymphatic system function and ISF drainage after SE.

### Post-SE cognitive impairment was improved after genetic deletion of Trpm4 and glibenclamide treatment.

**Figure 9:**
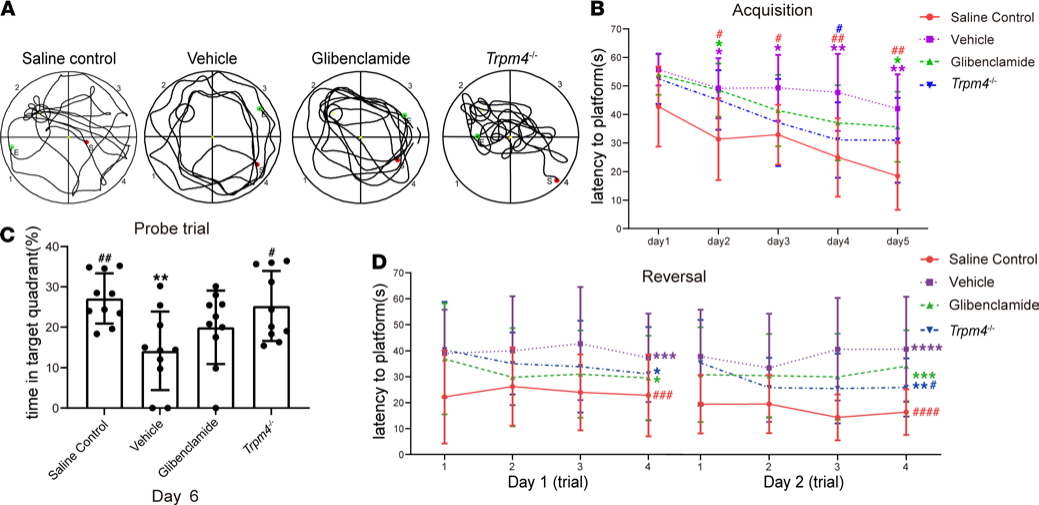
Genetic deletion of *Trpm4* and glibenclamide intervention improved cognitive function of mice 28 days after SE. (**A**) Representative swim paths of mice during the probe trial. (**B**) The latency to find the platform during acquisition after SE (*n* = 10 each). (**C**) The percentage of time spent in the target quadrant in the probe trial after SE (*n* = 10 each). (**D**) The latency to the platform in the reversal after SE (*n* = 10 each). Data are shown as the mean ± SD. Differences within multiple groups were examined by 1-way ANOVA with Bonferroni’s multiple comparison test. Continuous data were analyzed by 2-way ANOVA with Bonferroni’s multiple comparison test. *P < 0.05, **P < 0.01, ***P < 0.001, ****P < 0.0001 versus saline control group; ^#^*P* < 0.05, ^##^*P* < 0.01, ^###^*P* < 0.001, ^####^*P* < 0.0001 versus vehicle group.

We evaluated whether the earlier recovery of impaired glymphatic system function by deleting *Trpm4* and glibenclamide treatment improved cognitive function at 28 days after SE. Compared with the saline control mice, the results show that the overall performance of vehicle-treated mice in the Morris water maze (MWM) test was significantly decreased, presenting as increased latency to find the platform and decreased time in the target quadrant (Figure 9, A–D). In the MWM acquisition test, compared with the vehicle-treated mice, the latency to find the platform of glibenclamide-treated and *Trpm4^–/–^* mice displayed an improving trend. However, the result was not statistically significant. In the MWM probe trial and reversal test, *Trpm4^–/–^* mice showed decreased latency to find the platform and increased time in the target quadrant compared with the vehicle-treated mice (Figure 9, C and D; *P* = 0.0182 vs. vehicle group and *P* = 0.0118 vs. vehicle group). The glibenclamide-treated mice similarly showed a slight improvement. These data suggest that alleviation of cerebral edema by glibenclamide treatment or genetic deletion of *Trpm4* to recover glymphatic system function may help improve cognitive function after SE.

### p-Tau deposition and neuronal degeneration were attenuated after genetic deletion of Trpm4 and glibenclamide treatment.

**Figure 10:**
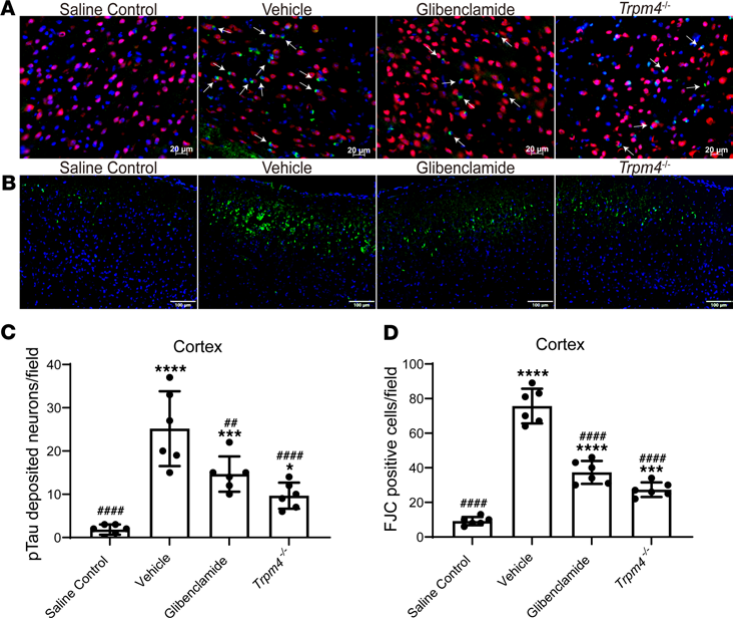
Genetic deletion of *Trpm4* and glibenclamide intervention alleviated p-tau–deposited neurons and neuronal degeneration of mice 28 days after SE. (**A**) Representative images of p-tau–deposited neurons (white arrows) in the cortex (red, NeuN; green, p-tau; blue, DAPI). Scale bars: 20 μm. (**B**) Representative images of degenerating neurons in the cortex (green, FJC; blue, DAPI). Scale bars: 100 μm. (**C**) Quantitative analysis of p-tau–deposited neurons that contain p-tau in the cortex (*n* = 6 each). (**D**) Quantitative analysis of FJC-positive cells in the cortex (*n* = 6 each). Data are shown as the mean ± SD. Differences within multiple groups were examined by 1-way ANOVA with Bonferroni’s multiple comparison test. Continuous data were analyzed by 2-way ANOVA with Bonferroni’s multiple comparison test. *P < 0.05,***P < 0.001, ****P < 0.0001 versus saline control group; ^##^*P* < 0.01, ^####^*P* < 0.0001 versus vehicle group.

Since the abnormal accumulation of ISF may lead to the deposition of toxic byproducts, we evaluated whether the deletion of *Trpm4* and glibenclamide treatment to reduce cerebral edema and promote ISF drainage are conducive to the elimination of p-tau (20), thus minimizing the neuronal damage caused by SE. The results showed that abundant p-tau–deposited neurons existed in the brains of vehicle-treated mice after SE (Figure 10A), while glibenclamide and *Trpm4* deletion significantly reduced the deposition of p-tau (Figure 10C. *P* < 0.05 vs. vehicle group). Moreover, compared with the vehicle-treated group, the number of degenerating neurons with positive fluoro-jade C (FJC) staining (21) in the parietal cortex (Figure 10B) was significantly decreased in the glibenclamide-treated group (Figure 10D; *P* < 0.0001 vs. vehicle group) and *Trpm4^–/–^* group (Figure 10D; *P* < 0.0001 vs. vehicle group). These results indicate that the improvement in glymphatic function after the alleviation of cerebral edema may eliminate metabolic waste and reduce brain histological injury after SE.

## Discussion

In this study, we found that glymphatic system function was temporarily impaired after SE. The time course of glymphatic system dysfunction matched well the progression of cerebral edema after SE. Furthermore, when cerebral edema was alleviated by glibenclamide treatment or genetic deletion of *Trpm4*, glymphatic system function recovered earlier, along with less deposition of p-tau and neuronal degeneration, and better cognitive function. These findings suggest 2 crucial concepts: cerebral edema after SE may lead to glymphatic system dysfunction, and the latter may be a key factor that renders the post-SE brain vulnerable to p-tau aggregation and the onset of neurocognitive impairment.

As an important structure mediating material exchange and waste removal in the brain, temporal regularity of glymphatic system dysfunction has been reported in various neurological injuries. In a mouse model of active immunization with ovalbumin, glymphatic system function declined 1 day after injury and returned to normal at 7 days (22), which was attributed to the obstruction of the PVS by immune complexes. In a mouse model of multiple microinfarcts, perivascular CSF tracer influx in the ipsilateral cortex was virtually abolished 3 days after injury. It was significantly reduced in the contralateral hemisphere and did not return to normal until 14 days after injury (23). Small, disperse ischemic lesions can impair brain-wide glymphatic function and impede waste removal in the area of the ischemic lesion, thereby promoting protein aggregation and neuroinflammation. After traumatic brain injury, CSF tracer influx was significantly impaired starting 1 day after injury, became the worst at 7 days, and remained impaired even at 28 days. This prolonged glymphatic function impairment contributes to the aberrant accumulation of p-tau (11). Similarly, through dynamic monitoring, we found for the first time to our knowledge that glymphatic system function began to decline starting 1 day after SE, reached the lowest point 3 days after SE, partially recovered 8 days after SE, and was associated with post-SE p-tau deposition and neuronal degeneration in the late phase. Notably, the time course of glymphatic system dysfunction matched well with the progression of post-SE cerebral edema, further supporting a previously reported but relatively unnoticed notion that persistence and spread of vasogenic edema may be related to an impairment or disruption of the normal perivascular fluid transport system of the brain (14). The association between cerebral edema and glymphatic system impairment also helps explain why acute post-SE injuries lead to chronic cognitive dysfunction.

Under normal circumstances, CSF moves along the PVS and flows into the brain parenchyma, driven by hydrostatic pressure or arterial pulsation, mixes with extracellular ISF and interstitial solutes such as p-tau, and eventually exits the brain along paravenous pathways. Following SE, seizures lead to blood-brain barrier (BBB) disruption and the consequent perivascular and parenchymal accumulation of serum proteins, followed by water influx into the PVS driven by increased osmolarity. Perivascular protein accumulation promotes water entry into the PVS and changes in the lipophilicity of the PVS (24), disturbing the liquid flow in the PVS. Cellular edema resulting from metabolic imbalance induced by abnormal neuronal activity also severely compresses the PVS and obstructs liquid flow in the PVS (13). In turn, glymphatic system dysfunction disrupts ISF removal and makes edema difficult to clear. Deletion of glial AQP4, which is critical for glymphatic system function, not only increases basal brain water content by increasing the ISF volume (25) but also leads to a higher rate of spontaneous hydrocephalus (26), indicating that disruption of ISF circulation may be a critical factor for edema formation and persistence. Meanwhile, disruption of ISF circulation would also favor the interstitial accumulation of waste products, sustain glial activation, cause neuroinflammation, and aggravate BBB disruption (27), which further exacerbate PVS obstruction and decrease ISF removal. Therefore, cerebral edema and glymphatic system dysfunction might form a vicious circle after SE. However, the current study failed to observe changes in cerebral edema after intervening in the glymphatic system due to technical reasons.

To explore the influence of cerebral edema on glymphatic system function, we used genetic deletion of *Trpm4* and glibenclamide treatment to reduce cerebral edema (5, 28). As expected, glymphatic system function recovered earlier, at 5 post-SE days, after intervention for cerebral edema, which further supports the assumption that a special connection exists between cerebral edema and glymphatic system dysfunction. Nevertheless, although cerebral edema was markedly alleviated 3 days after SE, we did not observe a parallel recovery of glymphatic function at this time point. This suggests that there may be more than one possible injury mechanism resulting in glymphatic system dysfunction. Similarly, in a mouse model of multiple microinfarcts, although only mild cerebral edema was formed in small disperse ischemic lesions, perivascular CSF tracer influx in the ipsilateral cortex was virtually abolished 3 days after SE, and accompanied by widespread reactive gliosis caused by inflammation in the ipsilateral cortex (23). Thus, perivascular inflammation may be an important factor that affects glymphatic function in addition to cerebral edema. After BBB disruption, the brain is susceptible to the incursion of circulating inflammatory factors (29). Additionally, the PVS acts as a crucial immune compartment where astrocytes, microglia, pericytes, endothelial cells, and leukocytes can interact directly (30) and produce proinflammatory cytokines such as VEGF-A, IL-1β, TNF-α, and IFN-γ after brain injury (31, 32). Although cerebral edema was alleviated, perivascular inflammation still existed 3 days after SE. This phenomenon is likely underlying the unrecovered PVS function at this time point.

The highly polarized expression of AQP4 water channels on astroglial end-feet is essential for efficient glymphatic function (8). We found that AQP4 expression and depolarization continued to increase after SE, and peaked 5 days after SE. The combined overexpression and depolarization of AQP4 was similar to that observed in an ALS model. This phenomenon was suspected to result from the increased abundance of AQP4 with wide distribution along with the astrocytic soma (33). Similarly to traumatic brain injury, sustained AQP4 depolarization may impair glymphatic function and hinder the clearance of interstitial solutes from the brain, leading to p-tau aggregation and the occurrence of neurodegeneration (11).

Recent findings have suggested that CSF surrounding the brain is the initial source of cerebral edema after cerebral ischemia (34, 35). CSF moves along the PVS and enters the brain and causes acute tissue swelling after middle cerebral artery occlusion, which is driven by initial ischemic spreading depolarizations result from the near-complete loss of cellular transmembrane ion gradients and subsequent vasoconstriction (36). However, this process only works at the very early stage of cerebral ischemia. Studies with longer observation times demonstrated reduced CSF influx within the PVS after ischemic stroke (12, 37). Unlike ischemic stroke, cerebral edema is undetectable in the very early stage (5 hours) after SE and usually becomes apparent after 1 day (38). Moreover, although seizures trigger cortical spreading depression, the latter only limits the generalization of seizures across the cortex by its electrical silencing effect rather than completely terminating SE (39). Indeed, our mice did not exhibit self-termination during SE. Therefore, CSF influx may not be the main source of cerebral edema after SE.

Regrettably, as stated above, although the improvement in glymphatic system function might alleviate cerebral edema, we have not found effective interventions to improve glymphatic system function after SE; therefore, there is a need to explore further the relationship between glymphatic system dysfunction and cerebral edema. Additionally, further studies should be conducted to explore their mechanism of interaction.

In summary, we demonstrated that glymphatic system function was impaired after SE and paralleled cerebral edema. Attenuation of cerebral edema was associated with earlier restoration of glymphatic function and cognitive function. The impairment of glymphatic system function involved in cerebral edema after SE may be a key factor that renders the post-SE brain vulnerable to p-tau aggregation and neurocognitive impairment. Given that a considerable proportion of patients have post-SE cognitive dysfunction, our results have important clinical implications, namely that targeting the glymphatic system by intervening in cerebral edema may represent a potential strategy for post-SE cognitive impairment. Moreover, as an FDA-approved drug, glibenclamide plays a dual role in reducing acute cerebral edema and improving long-term cognitive function in SE, which is worthy of clinical translational study in SE treatment.

## Methods

### Experimental design and animal preparation.

The present study included 2 parts (Figure 1A). In part 1, we observed the dynamic changes in glymphatic system function and cerebral edema, and the expression and polarization of AQP4, by histological examination within 8 days after SE. In part 2, we further explored the change in glymphatic system function, and p-tau aggregation, neuronal degeneration, and cognitive function, by using 2 methods, glibenclamide treatment or genetic knockout of *Trpm4*, that we previously demonstrated to alleviate brain edema after SE (5, 19).

Eight-week-old C57BL/6J male mice (22–25 g) were obtained from the Experimental Animal Center of Southern Medical University. In the second part, we used *Trpm4^−/−^* mice on a C57BL/6J background (Shanghai Model Organisms) and wild-type littermates propagated by homozygous mating. All mice were housed under a 12-hour light/12-hour dark cycle and had free access to water and standard chow throughout the experiments. All efforts were made to minimize the number of animals used and the pain inflicted on them.

### SE model and treatment.

The SE model was generated as described previously, with minor modifications (40). Briefly, lithium chloride (423 mg/kg, dissolved in normal saline; Sigma-Aldrich) was intraperitoneally administered 20 hours before pilocarpine (dissolved in normal saline; Sigma-Aldrich) injection, which was injected at 30 mg/kg first and 15 mg/kg 30 minutes later to induce SE. Methyl scopolamine nitrate (1 mg/kg, dissolved in normal saline; Shanghai Aladdin Biochemical Technology Co.,Ltd.) was injected 30 minutes before pilocarpine injection to reduce the peripheral effects of pilocarpine. A successful SE model was defined as the first-onset seizure with at least grade III on the Racine scale (41) lasting for 2.5 hours. Diazepam (20 mg/kg; Shanghai Aladdin Biochemical Technology Co., Ltd.) was administered 2.5 hours after SE onset with grade III. Saline control animals were injected with saline in place of pilocarpine.

To explore the correlation between glymphatic system function and cerebral edema, we used 2 kinds of intervention that were found to alleviate cerebral edema after SE in our previous studies (5, 19): glibenclamide treatment to pharmacologically block the unchecked opening of the sulfonylurea receptor 1–transient receptor potential M4 (SUR1-TRPM4) channel and *Trpm4^–/–^* mice to hinder the de novo assembly of the SUR1-TRPM4 channel. Glibenclamide (Sigma-Aldrich) was dissolved in dimethyl sulfoxide (DMSO) and then diluted in saline, with a final DMSO concentration of 0.05%. Mice in the glibenclamide group received intraperitoneal administration of glibenclamide at a loading dose of 10 mg/kg followed by 1.2 mg/6 hours for 3 days, while mice in the vehicle group received an equal volume of DMSO and saline (28).

### EEG.

EEG recordings were performed as described previously, with minor modifications (40). EEG was monitored by an MP150 system and analyzed with AcqKnowledge 5.0 software (BIOPAC Systems). The biopotential amplifier modules used a 3-electrode arrangement (VIN+, VIN–, and GND). The locations of the front 2 electrodes (VIN+, VIN–) were placed over the motor cortex (approximately 1 mm anterior to bregma and 1.5 mm lateral to the midline), and electrode GNDs were subcutaneously implanted in the mouse back. EEG data were recorded 1 hour before lithium chloride injection (baseline) until 1 hour after injection of diazepam.

### MRI experiments.

Brain MRI was performed as described previously, with minor modifications (28). The brains were scanned from the olfactory bulb to the brainstem by a 7.0 Tesla horizontal-bore magnet (PharmaScan 70/16 US, Bruker). T2WI and DWI method sequences were obtained. To measure the ADC, the cortex on a brain slice (bregma –2.30 to –2.46 mm) was manually drawn on T2WI maps as regions of interest. The mean value of ADC in the bilateral cortex of 6 animals per group was calculated using Image Display and Processing software (Bruker).

Contrast-enhanced MRI was used to visualize the brain-wide glymphatic system function in anesthetized mice. After T2WI and DWI scans, mice were scanned by contrast-enhanced T1-weighted imaging. The method was mostly performed according to previous publications (12). Six microliters of Gd-DTPA (469 mg/mL, MW 938 Da, Bayer) was microinjected into the cisterna magna at a rate of 1 μL/min through a PE-10 tube connected to Microliter Syringes (GaoGe) controlled by an ultramicro pump syringe (KD Scientific). After injection for 30 minutes, high-resolution T1-weighted imaging was obtained.

### Two-photon imaging of glymphatic system function.

Two-photon laser-scanning microscopy was used to visualize CSF influx into the brain parenchyma in anesthetized mice according to previous publications (42), with minor modifications. Mice were anesthetized by intraperitoneal injection of chloral hydrate (4.2%, dissolved in saline, 0.01 ml/g; Aladdin). A thin cranial window (3 mm in diameter) was made over the cortex to visualize the vasculature and PVS. Six microliters of fluorescein isothiocyanate–dextran (FITC-dextran; 70 kDa, 1% dissolved in artificial CSF; Sigma-Aldrich) was microinjected into the cisterna magna to evaluate glymphatic system function 30 minutes before imaging (Figure 2A). Rhodamine B isothiocyanate–dextran (RITC-dextran; 0.2 mL, 70 kDa, 1% dissolved in saline; Sigma-Aldrich) was injected intravenously to visualize the vasculature immediately before imaging. An Olympus 2-photon imaging system (Fluoview FV1200) equipped with a water immersion objective (25×, 1.05 NA) was used for imaging. Images of the XYZ stacks (512 × 512 pixels, 1 μm resolution) were synthesized as pseudo–3-dimensional (pseudo-3D) images by the Olympus Viewer software.

### Ex vivo imaging of the influx function of the glymphatic system.

To further evaluate brain-wide glymphatic system function, following the method described previously (43), 6 μL of 70 kDa TMRE-dextran (1% dissolved in artificial CSF; Thermo Fisher Scientific) was microinjected into the cisterna magna. After TMRE-dextran injection for 30 minutes, mice were euthanized and transcardially perfused with PBS to clear blood and then with PFA to fix tissue (4% dissolved in PBS). The brain was removed and immersed at 4°C, postfixed for an additional 24 hours in 4% PFA, rinsed, and stored in PBS before sectioning. Brain sections (100 μm thick) limited by bregma −1.00 mm (rostral) and bregma −2.00 mm (caudal) were cut on a vibratome (Leica VT1000S). Sections were imaged on an Olympus FV10i confocal microscope.

### Ex vivo imaging of efflux of ISF.

Interstitial solute drainage from the brain into dCLNs was observed after SE following a method described previously (44), with minor modifications. Two microliters of Alexa Fluor 555–ovalbumin (OVA-555; 0.5 mg/mL in artificial CSF; Thermo Fisher Scientific, O34782) was stereotactically injected into the parenchyma within 10 minutes at the following coordinates: 1.5 mm anterior/posterior (AP), 1.5 mm medial/lateral (ML), and –2.5 mm dorsal/ventral (DV) from bregma (Figure 5A). After injection for 2 hours, dCLNs were dissected alive, and mice were perfused and fixed for brain specimens. The dCLNs were placed on a slide and observed using an Olympus IX73 inverted microscope. The brains were postfixed and cut into 100 μm thick slices via vibratome. The images were acquired with an Olympus stereo microscope and analyzed with ImageJ software (NIH) by a blinded investigator.

### Assessment of post-SE cognitive impairment.

Cognitive function was assessed by the MWM test 28 days after SE. The MWM test was performed as described previously (5). All mice were transported to the behavior room to habituate for at least 24 hours before testing. The MWM test consisted of 5 days of acquisition, 1 day of probe trial, and 2 days of reversal. All MWM testing was performed between 12:00 and 17:00 hours, during the lights-on phase, by a blinded experimenter. During the acquisition, probe, and reversal, data were recorded using an animal behavior analysis system (VIDEOMOT 2, TSE Systems).

### Histological examination.

The mice were euthanized and transcardially perfused with ice-cold PBS, followed by PFA for brain fixation. The brain was postfixed in PFA for 24 hours and then rinsed and gradient dehydrated by 20% and 30% sucrose dissolved in PBS. Coronal brain sections (10 μm thick) limited by bregma −1.00 mm (rostral) and bregma −2.00 mm (caudal) were cut on a Cryostat Microtome (Leica CM1800). For immunofluorescent staining, the sections were mounted onto slides, permeabilized with 0.3% Triton X-100, and then blocked with 10% goat serum. Then, the sections were incubated overnight at 4°C with a primary antibody (1:100 anti-AQP4 antibody, Proteintech, 16473-1-AP; 1:100 anti–glial fibrillary acidic protein [anti-GFAP] antibody, Abcam, ab10062; 1:100 anti-tau antibody, Abcam, ab92676; 1:100 anti-NeuN antibody, Abcam, ab104224). After washing away the primary antibodies, the sections were incubated with secondary antibodies (1:200 goat anti–rabbit IgG H&L [Alexa Fluor 488], Abcam, ab150081; goat anti–mouse IgG H&L [Alexa Fluor 594], Abcam, ab150120). Finally, the sections were embedded in Fluoroshield mounting medium with DAPI (Abcam, ab104139), and covered with a coverslip. According to the reagent instructions, degenerating neurons were detected using fluoro-jade C (FJC) staining (Biosensis, TR-100-FJT). AQP4 expression and polarization were evaluated as described previously (45). The higher the AQP4 polarity was, the greater the proportion of immunoreactivity restricted to perivascular regions, while the lower the AQP4 polarity was, the more evenly the immunoreactivity distributed between the perivascular end-feet and the soma (Figure 6B). Protein expression and AQP4 polarization were evaluated and analyzed by an experimenter blinded to group allocation using Image J.

### Statistics.

All statistical analyses were performed using GraphPad Prism 8. Data are presented as the mean ± SD. Differences within multiple groups were examined by 1-way ANOVA with Bonferroni’s multiple comparison test. Continuous data were analyzed by 2-way ANOVA with Bonferroni’s multiple comparison test. Results with a *P* value of less than 0.05 were considered significant.

### Study approval.

This study was approved by the Animal Care and Use Committee of Nanfang Hospital, Southern Medical University (NFYY-2018-115) and complied with the National Guidelines for Animal Experimentation (Guidelines on Administration of Laboratory Animals in China and Guidelines on the Humane Treatment of Laboratory Animals in China) and the Animal Research: Reporting of In Vivo Experiments (ARRIVE) guidelines.

## Author contributions

KL and JZ performed animal experiments. KL wrote most of the manuscript. YC prepared and modified figures. ZS, ZL, XC, XL, and CL provided technical support. KH guided experiments and revised the manuscript. SP designed the study and modified the manuscript. All authors read and approved the final manuscript.

## Version 1 09/08/2021

Electronic publication
